# Effect of Nanometric Particles of Bentonite on the Mechanical Properties of a Thermoset Polymeric Matrix Reinforced with Hemp Fibers

**DOI:** 10.3390/polym15061571

**Published:** 2023-03-22

**Authors:** Meylí Valin Fernández, María José Ahumada González, Rolando Briones Oyanadel, José Luis Valin Rivera, Angel Rodríguez Soto, Alvaro González Ortega, Cristobal Galleguillos Ketterer, Alexander Alfonso Alvarez, Francisco Rolando Valenzuela Diaz, Gilberto García del Pino

**Affiliations:** 1 Department of Mechanical Engineering (DIM), Faculty of Engineering (FI), University of Concepción, Concepción 4030000, Chile; 2 Escuela de Ingeniería Mecánica, Pontificia Universidad Católica de Valparaíso, Valparaíso 2340025, Chile; 3 Departamento de Ingeniería Mecánica, Facultad de Ingeniería, Universidad de la Serena, La Serena 1720010, Chile; 4 Departamento de Engenharia Metalúrgica e de Materiais, Escola Politécnica, Universidade de São Paulo (USP), São Paulo 05508-220, Brazil; 5 College of Technology, State University of Amazonas, Manaus 69850-000, Brazil

**Keywords:** vegetable fiber, bentonite, polymer composite, mechanical properties

## Abstract

The influence of the addition of bentonite nanoparticles on the tensile and flexural strength of a thermosetting polymer matrix composite material reinforced with hemp fibers was de-terminated. All composites were manufactured with 5% of bentonite in the polymer mass–weight ratios and 10 to 45 wt% of fibers with a step of 5%. For mechanical characterization, tensile and flexural tests were performed: scanning electron microscopy and energy-dispersive X-ray spectroscopy analyses were carried out. The tensile strength of the samples containing bentonite compared to the polymer samples with the fiber addition was affected for all fiber addition percentages, except for 35% while the flexural resistance improved with the addition of bentonite in the percentages of 20, 30, 35, and 45% of fiber addition. With the addition of bentonite, the maximum values of tensile and flexural strength were both obtained for the 35% addition of fibers, with values of 34.28 MPa and 98.04 MPa, respectively. The presence of bentonite favored the rigidity of the material to traction and bending, which was reflected through an increase in the elastic modulus compared to the composite that only had fiber. The maximum values obtained were 9065 MPa in tension and 8453 MPa in flexion for the 40% and 35% of addition of fiber, respectively. Microscopy showed a good distribution of fibers in the matrix, the absence of internal porosities, and a good interaction between matrix and reinforcement.

## 1. Introduction

Reinforced polymer composites are the base of many modern products, since due to the combination of properties, they report a better mechanical and economical response [1,2,3]. The applications of the composites are diverse in different areas, marine [4], automotive [5], civil infrastructure [6], and biomedical [7]. With the level of industrialization and pollution existing worldwide, it is necessary to think about materials that cause low environment contamination. One option is to replace conventional synthetic reinforcements with vegetable fiber reinforcements, which in addition to not interfering with the environment, have a lower manufacturing cost, low weight, biodegradable, and good mechanical properties [8].

Suriyaprakash et al. presented a mechanical characterization of hybrid epoxy composites reinforced with ramie particles, hemp fibers, and coconut shell, laminated by compression molding where the best results in mechanical properties were found in the compositions with higher percentages of hemp (14%), with values of 71.5 MPa for the tensile strength and 104.7 MPa for the flexural strength [9]. Jute fiber, on the other hand, was studied as reinforcement by a vacuum-assisted epoxy resin infusion with different fiber orientations by Hossain et al. [10], with mechanical characterization by three-point tensile and flexural tests, showing results of higher strength in the longitudinal direction than in the transverse, with a value of 39.10 MPa for a 0–90 laminate configuration. A combination of unsaturated polyester resins such as a matrix with sisal, nacha, and e-glass fiber as reinforcements were used by Miliket et al. [11] as a hybrid composite for wind turbine blade applications using manual lamination, with a contribution to the tensile strength of 220.12 MPa (10 wt% of glass, 10 wt% of nacha, and 20 wt% sisal), compressive strength of 308.55 MPa (10 wt% of glass, 5 wt% of nacha, and 15 wt% sisal), and flexural strength of 210.43 MPa (10 wt% of glass, 10 wt% of nacha, and 20 wt% sisal). Otherwise, Mahesh et al. [12] studied the potential of a hybrid composite of pure polypropylene, sisal fibers, ramie fibers, and maleic grafted anhydride (MagH) developed by the melt blending method with the aid of twin-screw extrusion molding with the maximum value of the tensile strength of 41.2 MPa (87 wt% of polypropylene, 3 wt% of MagH, 5 wt% of sisal, and 5 wt% of ramie fibers) and flexural strength of 60.5 MPa (67 wt% of polypropylene, 3 wt% of MagH, 15 wt% of sisal, and 15 wt% of ramie fibers). Banana, coconut, jute, and cotton fibers as reinforcement at different weight ratios with a matrix of polyethylene were also studied by Paladugu et al. [13], with the maximum value of the tensile strength of 55 MPa (70 wt% of matrix, 5 wt% of jute, 5 wt% of banana, and 20 wt% of coconut) and a flexural strength of 1700 MPa (70 wt% of matrix, 5 wt% of cotton, 18 wt% of banana, and 7 wt% of coconut).

In Chile, a plant species that is of interest is hemp (Cannabis sativa), whose cultivation in the country is used mostly in the textile industry today. Hemp fibers, as a reinforcement of polymeric matrix composite materials, have reported improvements in mechanical properties such as longitudinal and transverse Young’s modulus as well as the shear Young’s modulus [14]. On the other hand, Panaitescu et al. [15] exposed in their work a polymer matrix composite reinforced with hemp fiber at lengths of 1, 2.5, and 4 mm obtained through an extrusion process where the maximum value of resistance to tensile strength was 39 MPa (polypropylene, 5 wt% of maleic anhydride grafted polypropylene, 15 wt% of Kraton 1652 G, 30 wt% of hemp fiber with 4 mm of length) and a tensile Young’s modulus of 3000 MPa. Neves et al. [16] conducted a study where they manufactured two composites: one with a base of epoxy and hemp fibers and the other with a base of polyester resin and hemp fibers. The flexural strength maximum value of 76.69 MPa was obtained for the 30 wt% of hemp fiber with epoxy, compared with the value of 50.75 MPa for the hemp fiber and polyester resin. For the tensile strength, the maximum value of 50.46 MPa was also obtained for the 30 wt% of hemp fiber with epoxy compared with the value of 31.46 MPa for hemp fiber and polyester resin.

Another way to modify the properties of the resin is by adding particles, which depends on the configuration and interfacial interaction of the latter with the resin [14]. Bentonite, for example, is a type of clay that has good water absorption and compressive strength behavior [16,17]; good sound absorption capacity in combination with natural fibers [18], or impermeability under high concentration of heavy metal solution [19]. Its contribution in the improvement in mechanical properties has been reflected by Arash et al. [20], in combination with polylactic acid polymer and thermoplastic polyurethane where the Young’s modulus in tension was 1650 MPa for a 3 wt% of bentonite particles compared with the 15,545 MPa of the thermoplastic polyurethane; in combination with epoxy resin as presented by Mahadeva et al., where the tensile and compressive strength were 18 MPa (7 wt% of nanoparticles of bentonite) and 190 MPa (7 wt% of nanoparticles of bentonite) compared with 14 MPa and 100 MPa of the epoxy resin only [21]; in combination with polystyrene where the alignment of the clay particles into the polymer matrix reduced the resistance compared to the composite for a random orientation of the clay particles [22].

In the present work, the influence of nanometric bentonite particles in the mechanical properties of a thermoset polymer matrix composite material reinforced with hemp fibers was carried out. In the present work, the influence of nanometric bentonite particles on the mechanical properties of a thermoset polymer matrix composite material reinforced with hemp fibers was studied. The novelty is reflected through the following points:

▪ The literature consulted thus far does not report work with these materials and the manufacturing method proposed here.
▪ It was possible to verify that nanometric-sized bentonite particles guarantee a better interaction with the matrix.
▪ The use of nanometric bentonite particles improves the stiffness of the compound by increasing its tensile and flexural elastic modulus.

## 2. Materials and Methods

### 2.1. Materials

An orthophthalic unsaturated polyester polymer (CRISTALÁN 859) was selected and its properties are shown in Table 1. Methyl ethyl ketone peroxide (MEK) (with 9% active O_2_) and cobalt octoate (6% Co) were used as a catalyst and as an accelerator for hardening at room temperature, respectively, manufactured by Andercol S.A., Colombia. It was determined that the best proportion of the elements for the 2% catalyst was between 0.05% and 0.1% of the accelerator (based on the mass of resin used for each plate).

The hemp fibers used as reinforcement were obtained from commercial braided hemp, which was separated into strands. The bentonite used as the ceramic filler was Rheotix VP bentonite of the calcium type provided by the University of Sao Paulo (USP).

### 2.2. Fabrication of the Composite

The fiber length was determined according to the critical length criterion, as presented by Equation (1) [23].

(1) $$l_{c}=\frac{\sigma_{f}d_{f}}{2\tau_{c}}$$

where *σ_f_* is the maximum stress that the fiber resists; *d_f_* is the fiber diameter; *τ_c_* is the shear strength of the matrix; and *l_c_* is the critical length.

First, the diameter of the fibers was measured, and for this purpose, a Carl Zeiss Jena optical microscope at 100× coupled to a computer was used. Subsequently, the maximum stress that the fiber resists and the shear stress were determined using a universal testing machine model WDW-200E from the TIME Group Inc., with a maximum load of 200 kN and a precision of ±0.0001 kN for a total of 20 fibers. With the results obtained, the fiber diameter (0.016 mm), the ultimate stress (260.4 MPa), and the shear stress of the matrix (4.3 MPa), the length value obtained was 4.8 mm, so it was decided to cut the fiber with a length of 25 mm.

For the manufacture of the composite material, three box-type molds of SAE 1045 material were used, stacked one on top of the other, which allowed for the manufacture of several plates at the same time by the molding compression method, making it possible to obtain composite plates with the dimensions of 250 × 156 × 3 (mm).

For the tensile strength analysis, only fiber boards were manufactured, in percentages of weight ratios from 10 to 45 wt% of hemp fiber in five steps with random orientation in proportion to the mass of the polymer. For both the analyses of the tensile and flexural tests, plates with the same proportions above-mentioned were prepared with the addition of bentonite, which was set at a value of 5 wt% in proportion to the mass of the polymer. The bentonite nanometric particles were fixed to 5 wt% based on the polymer mass according to the results of Fernández et al. [23]. All of the plates were made by mixing the materials manually and once incorporated into the molds, they were covered and taken to a press that applied a load of 4 tons for a period of 24 h. Once removed from the molds, they were stored and protected from light for 25 days to comply with the post-curing process.

### 2.3. Mechanical Characterization

**Figure 1:**
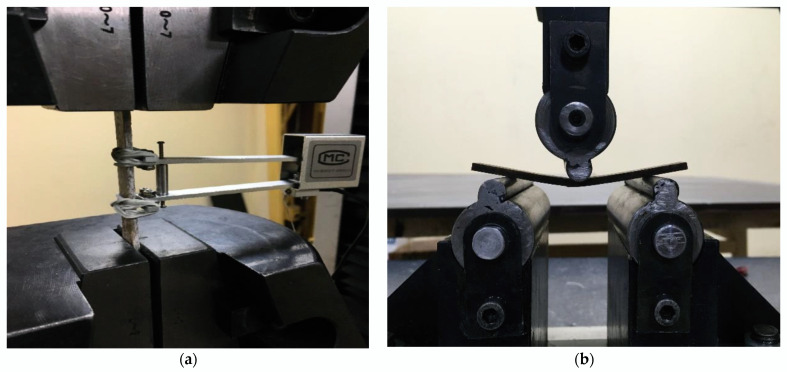
Experimental tests: (**a**) tensile test and (**b**) flexural test.

The mechanical characterization was carried out through tensile and bending tests. The tensile tests were carried out following the ASTM D638-14 standard in a universal testing machine model WDW-200E from the TIME Group Inc., with a maximum load of 200 kN and a precision of ±0.0001 kN; a model MC extensometer and a precision of 0.001 were used under a monotonic load over time and with a strain rate of 1 mm/min, as presented in Figure 1a. To obtain the geometry indicated by the standard, the plates were cut using a BCL 1309X model laser from the BODOR company with an intensity of 10 (mA) and a cutting speed of 14 mm/s at a distance of 3 mm from the nozzle of the machine to the surface of the plate to be cut. Once the rectangles were obtained to obtain the neck of the test tubes, a model CAMM-3 PNC-2500 milling cutter from the company Roland Digital Group was used. For this operation, a 6 mm diameter milling cutter with a rotation speed of 6000 rpm and a linear speed of 14 mm/s was used, with successive passes at 3.6 mm from one to the other. The dimensions of the tensile specimen were 165 × 19 × 3 (mm).

The bending tests were carried out following the ASTM D7264-15 standard in the universal machine described above, with the same extensometer and using the same speed, with three support points, as presented in Figure 1b. The specimens, being rectangular, were cut by laser, with the same equipment used for the manufacture of tensile specimens and maintaining the same cutting parameters. The dimensions of the bending specimen were 96 × 13 × 3 (mm). The nomenclature used for the samples is presented in the Table 2.

An analysis was carried out by scanning electron microscopy (SEM) using ZEISS EVO MA10 equipment to obtain information on the internal structure of the hemp stem such as the thickness, cell wall area, morphology, and in the case of the fractures, observe the interface between the polymer and the fiber. The microscopies in the fractures of the material were carried out in tensile specimens, and as it is a non-conductive material, they were metallized with a thin layer of gold.

On the other hand, energy-dispersive X-ray spectroscopy (EDS) analysis was carried out on the bentonite to obtain its characterization, both in terms of the morphology and chemical composition. For this, a Hitachi SU3500 model scanning electron microscope was used with an XFlash 410-M.

## 3. Results and Discussion

### 3.1. Energy-Dispersive X-ray Spectroscopy (EDS)

**Figure 2:**
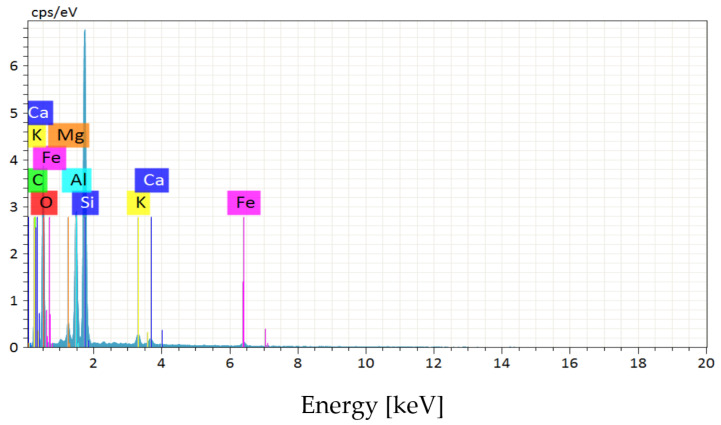
EDS analysis.

For the EDS analysis, three samples of dry untreated bentonite were used. Figure 2 shows the diffractogram of a bentonite sample, and Table 3 shows the chemical composition.

Table 3 shows that the sample contained 0.66% calcium, which is why it is classified as a calcium-type bentonite. Other important components found were carbon, silicon, and aluminum.

### 3.2. Scanning Electron Microscopy

**Figure 3:**
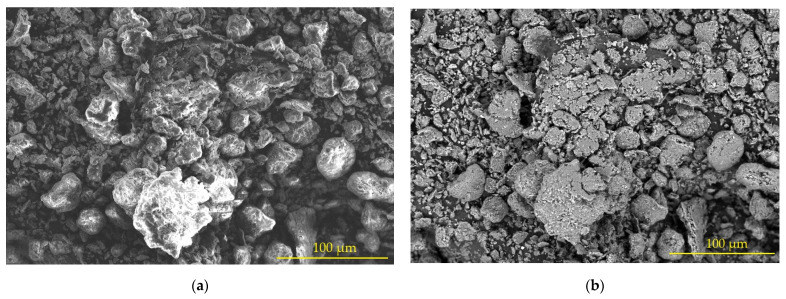
Scanning electron microscopy (SEM) in a bentonite sample: (**a**) variability of grain size; (**b**) surface appearance.

From the SEM analysis of the bentonite samples, the images presented in Figure 3 were obtained. It can be seen in Figure 3a that the size of the grains varied in the sample, perceiving in the center of this a grain of greater dimension with respect to those that surrounded them. Figure 3b shows that the irregular surface has features that are suitable for the interface (adhesion) between the polyester resin and the bentonite, as presented by Seghar and Azem [24]. The variation in the particle size ensures better interaction with the polymer matrix, where smaller grains provide better results when the material is subjected to traction due to the increase in the surface area of the particle size, which increases the rate of attraction of particles in the bentonite to polyester polymer [25].

The grains have, in general, a shape with a tendency to sphericity. This favors the active surface (particle–matrix interface) and slows down the propagation of cracks (decreased stresses). However, it has been found that elongated shapes, a “needle shape”, offer better mechanical properties [26,27].

**Figure 4:**
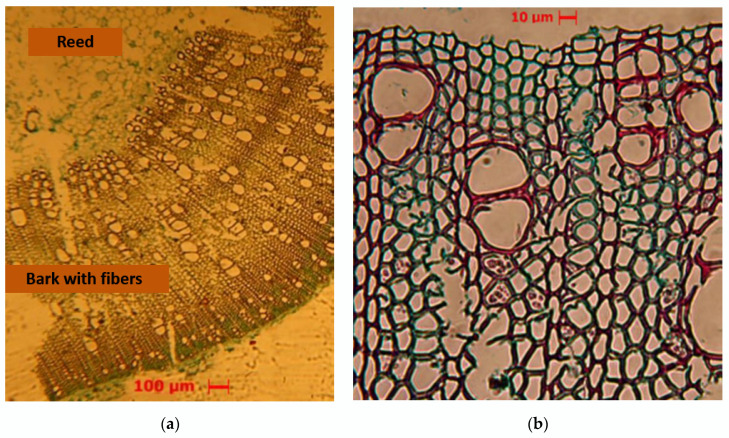
Optical microscopy in a sample of hemp: (**a**) cross section in the stem, 40×; (**b**) cells that make up the fiber, 400×.

Optical microscopies of the cross section of hemp stems were obtained where the shape of the fibers, the thickness of the cell wall, and its area can be appreciated. These factors influence the strength of the fibers and their structural function as reinforcements in the composite. Figure 4a shows the hue of the stem and the softer substance called the reed characteristics that contribute to thermal and acoustic insulation, while Figure 4b depicts the cells that make up the vegetable fiber.

In natural fibers, there is, in general, disparity in the shape and size of the cross section, however, in Table 4, both the thickness of the wall of the fibers and the area of said wall in narrow margins could be observed.

**Figure 5:**
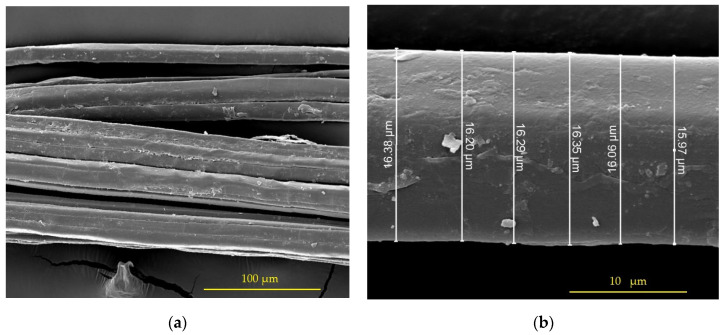
Scanning electron microscopy (SEM) in the hemp sample: (**a**) bundle of fibers, 1000×; (**b**) measurements of a fiber, 10,000×.

Figure 5 shows the diameter of the fiber, which can also be perceived as having a rough surface; this condition improves the union between the polymer and the fiber.

**Figure 6:**
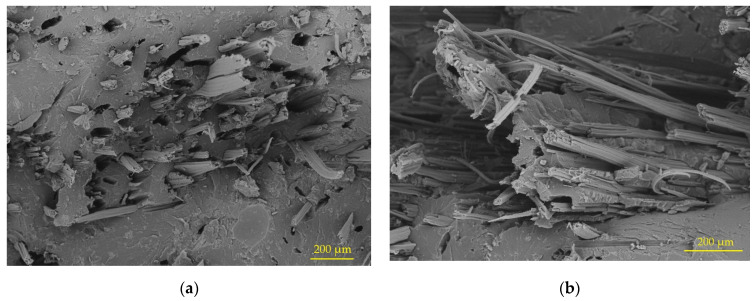
Scanning electron microscopy (SEM) in the tensile test specimen fracture zone: (**a**) sample PPH25, 146×; (**b**) sample PPH20, 200×.

Additionally, once the tensile tests were carried out, an analysis of the fracture zones was executed. Figure 6 shows the breakage areas of the test tubes identifying the separation of the fibers from the matrix, breakage of the fibers, and breakage of the polymeric matrix. We also observed the topography of each fiber, and the irregularities that favor the mechanical union of the interface. The presence of air bubbles or the inclusion of other defects that affect the resistance of the compound was not detected.

### 3.3. Tensile Strength

**Figure 7:**
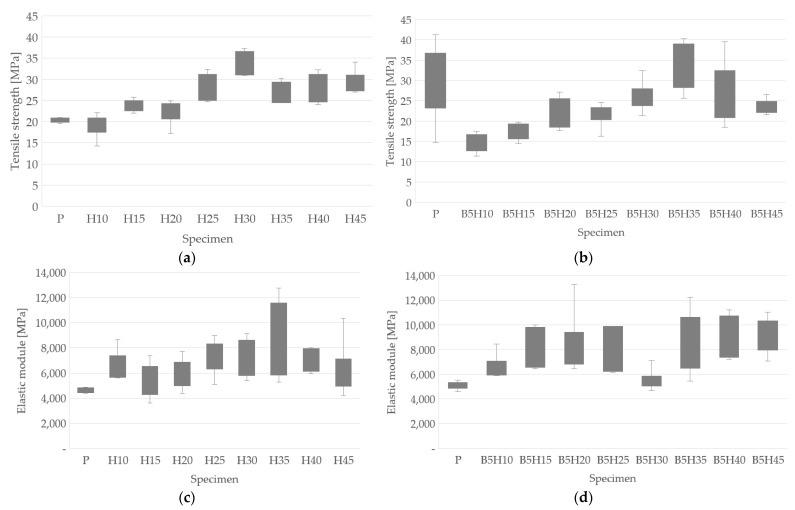
Tensile test results: (**a**) tensile strength of samples with addition of fibers only; (**b**) tensile strength of samples with addition of fibers and bentonite; (**c**) elastic module of samples with addition of fibers only; (**d**) elastic module of samples with addition of fibers and bentonite.

The tensile test was carried out on a quantity of not less than five test pieces of each of the percentages indicated above. The tensile strength values for the samples with the addition of fiber only and the samples with the addition of fiber and bentonite are shown in Figure 7a and Figure 7b, respectively. In Figure 7c,d, the values of the elastic module are presented for the samples with the addition of fiber only and the samples with the addition of fiber and bentonite, respectively.

The specimens without fibers behaved like a brittle material, the stress grew constantly up to the point of fracture, and there was no point at which the slope changes, differentiating between the elastic and plastic zones; there was only an elastic zone. The tensile strength was the same as the rupture stress and the dispersion was low. For the rest of the samples, a certain degree of curvature was observed due to the influence of the reinforcement (lignocellulosic fibers, with a viscoelastic behavior), but the yield point could not be clearly defined; however, by applying the 0.2% criterion, it was verified that there was no point that divided the elastic and plastic behavior, so the material presented only the elastic zone. As the percentage of fiber increases, the tensile strength increases up to 30 wt% of fiber addition, and then began to decrease. This is a trend in fiber-reinforced polymer matrix composite materials, and it is justified because there is a limit to the amount of fibers that can begin to optimally cohere the matrix; when this limit is exceeded, a decline in the properties of the material occurs, as presented by Vallejos et al. [28] and Rodriguez et al. [29].

For the specimens with the addition of fibers and bentonite, the tensile strength decreased in all of the samples, except for B5H5, which presented an increase of 26.96% with respect to sample H5, as can be seen in Figure 7a,b. This decrease in tensile strength due to the presence of bentonite may be due to the agglomerations of the particles within the matrix. Small aggregates that form act as stress concentrators, acting as failure initiators. The increase in the tensile strength presented by B5H5 could be attributed to the rigidity of the filler particles. A similar behavior was reported by Sarkar et al. [27], where resistance presents a turning point in their results.

By also comparing the results between the compound with and without bentonite, an increase in the dispersion of the values was found. This can be proven by the random distribution of this within the matrix, which can generate a different mechanical response for each sample, and a similar behavior was observed by Ollier et al. [28].

Table 5 shows a summary of the experimental results for the elastic modulus and the maximum tensile strength. Both are presented as an average value, which was obtained by adding all the tests carried out for the same specimen and dividing this value by the number of tests carried out on it. A comparison was made between the samples with and without the addition of bentonite, defining the increase/decrease in the elastic modulus and the maximum tensile strength.

The elastic modulus of the samples fabricated with the polymer and fibers did not present a defined progressive increase, but there was an increase in its value as the percentage of fibers in the sample increased, where the best result of 8384 MPa was obtained for H35.

The presence of bentonite contributed rigidity to the composite, and this could be observed in the comparison between the pairs with and without the addition of bentonite, where increases were perceived for almost all the combinations, except for the B5H30 samples, which showed a decrease of 23.06% compared to their peer H30. This result may also have been influenced by the size of the bentonite particles, where authors such as Seghar and Azem [24] also found that a small particle size increased the rigidity of the material, while a larger one would favor the formation of agglomerates in the matrix. On the other hand, it was possible to define that the tensile strength only increased in the B5H35 samples with an increase of 26.96 % compared to its peer H35. This reduction in mechanical resistance may be due to the agglomeration of the nanometric bentonite particles, which may have led to poor dispersion in the polymer matrix. A similar behavior was presented by Bahari et al. [30] and Khandelwal et al. [31].

### 3.4. Flexural Strength

**Figure 8:**
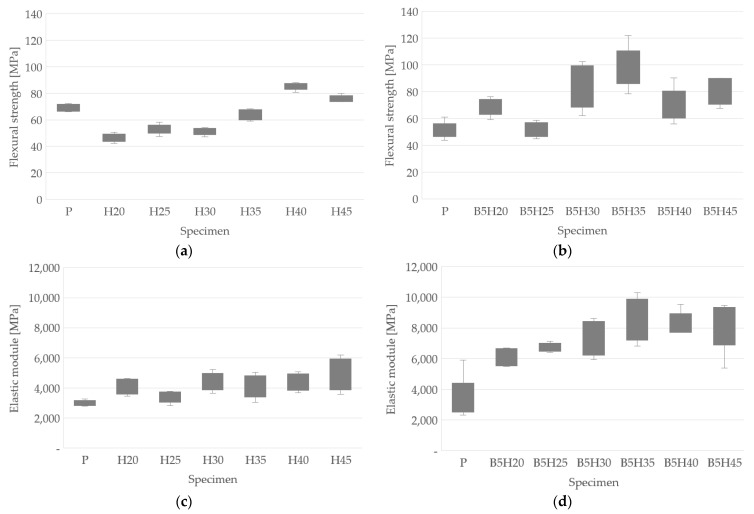
Flexural test results: (**a**) flexural strength of samples with the addition of fibers only; (**b**) flexural strength of samples with the addition of fibers and bentonite; (**c**) elastic module of samples with the addition of fibers only; (**d**) elastic module of samples with the addition of fibers and bentonite.

Figure 8 presents the flexural test results for all species. Figure 8a shows the flexural strength for the samples with the addition of fiber only, whereas Figure 8b shows the flexural strength for the samples with the addition of fibers and bentonite. On the other hand, in Figure 8c,d the elastic module for the samples with the addition of fiber only and the samples with the addition of fibers and bentonite are presented, respectively. For each specie, no less than five tests were carried out.

In the samples manufactured only with the addition of fibers, the flexural resistance only improved in the H40 and H45 samples with 85.22 MPa and 76.06 MPa, respectively, compared to the results obtained for the polymer sample P, which was 69.05 MPa, as can be seen in Figure 8a. The maximum value was found for the H40 sample. The presence of bentonite improved the flexural strength in samples B5H20 (68.92 MPa), B5H30 (83.51 MPa), B5H35 (98.04 MPa), and B5H45 (79.55 MPa) with increases of 49.44, 63.26, 54.50, and 4.59%, respectively, with respect to their pairs H20 (46.12 MPa), H30 (51.15 MPa), H35 (63.87 MPa), and H45 (76.06 MPa) and the polymer sample P (68.05 MPa), as can be seen in Figure 8b. Similar results were found by Rapacz-Kmita et al. [32].

The elastic modulus, on the other hand, improved in all of the samples with the addition of bentonite compared to their pairs containing the fiber and polyester polymer. The best result was obtained in the B5H35 (8453 MPa) samples with an increase of 139.26% compared to its pair B5H35 (35.33 MPa). The presence of 5 wt% of bentonite improved both the resistance and the elastic modulus when compared with the specimens that contained polymer and fibers, which agrees with the results obtained by [28], where these improvements were perceived in a compound based on polymer and bentonite. A summary of the results with the average values of the elastic modulus and the flexural strength are presented in Table 6.

## 4. Conclusions

A novel composite material with a polyester polymer matrix reinforced with hemp fibers and bentonite particles was manufactured, where the influence of the latter on the mechanical properties such as tensile and flexural strength was determined. The percentage of bentonite was set at 5%, while that of the hemp fibers ranged from 0% to 45%, and the following conclusions can be drawn:

1. Bentonite clay improved the rigidity of the material, reporting an increase in the elastic modulus, whose maximum value of 7938 MPa was found in the sample that had an addition of 15% of fibers, which represents an increase of 45.01% with that compared to its pair, which was made up of polyester polymer and fibers.
2. The tensile strength was affected by the addition of bentonite and all of the samples showed a decrease compared to their peers. Except for the B5H35 sample, which presented a value of 34.38 MPa, an increase of 26.96% with respect to its pair made of polyester polymer and fibers.
3. The flexural elastic modulus improved in all of the samples with the addition of bentonite when compared with similar ones that had reinforcing fibers. The best result of 8453 MPa with an increase of 139.26% was found for a combination of the 35% addition of hemp fibers (B5H35 sample).
4. Flexural strength showed improvements when bentonite was added, except for cases where it was combined with the 25 and 40% addition of hemp fibers. The maximum value found of 83.51 MPa, which represents an increase of 63.26% with respect to its pair composed of polyester polymer and hemp fibers, was found for a 30% addition of fibers.
5. For all materials manufactured, either with the addition of fibers alone or with the addition of fibers and bentonites, a good distribution of the fibers in the matrix was achieved, and a good adhesion between the matrix and the fibers as well as the absence of defects.

## Figures and Tables

**Table 1:** The properties of Cristalán 859.

Properties	Typical Values
APHA Color	80 maximum
Acid number	32 maximum
Brookfield viscosity (cps), 25 °C (needle 2, 20 r.p.m., 1 min).	500–700
Solid (%)	64–66
Gel time at 25 °C, min.	7–10
Reactivity:	
Exotherm temperature, °C.	140–160
Exotherm temperature, min.	17–25
Stability at 60 °C, horas.	168 minimum

**Table 2:** Nomenclature of the samples.

Specimen	Polyester Polymer (P) [%]	Hemp Fiber (H) [%]	Bentonite (B) [%]
P	100	-	-
H10	90	10	-
H15	85	15	-
H20	80	20	-
H25	75	25	-
H30	70	30	-
H35	65	35	-
H40	60	40	-
H45	55	45	-
B5H10	85	10	5
B5H15	80	15	5
B5H20	75	20	5
B5H25	70	25	5
B5H30	65	30	5
B5H35	60	35	5
B5H40	55	40	5
B5H45	50	45	5

**Table 3:** Bentonite chemical composition.

Element	At. No.	Netto	Mass [%]	Mass Norm. [%]	Atom [%]	Abs. Error [%] (1 Sigma)	Real. Error [%] (1 Sigma)
Oxygen	8	7219	40.28	45.00	47.12	5.95	14.77
Carbon	6	1609	22.90	25.59	35.69	4.60	20.08
Silicon	14	18,146	15.47	17.28	10.31	0.70	4.55
Aluminum	13	6830	7.04	7.87	4.89	0.38	5.46
Magnesium	12	886	1.12	1.25	0.86	0.11	9.61
Iron	26	366	1.06	1.19	0.36	0.09	8.59
Potassium	19	792	0.96	1.08	0.46	0.07	7.69
Calcium	20	459	0.66	0.74	0.31	0.06	9.76
		Sum.	89.50	100.00	100.00		

**Table 4:** Thickness and area of the cell wall of hemp.

Specie	Sample	Cell Wall Thickness [μm]	Cell Wall Area [μm^2^]
Hemp	1	1.9	49.5
	2	2.0	55.9
	3	2.1	54
	4	1.9	55
	5	2.1	53
	Average	2.0	53.5

**Table 5:** Summary of the results of the tensile test.

Specimen	Elastic Module [MPa]	Increase [%]	Tensile Strength [MPa]	Increase [%]
P	4674	-	20.2	-
H10	6373	-	19.11	-
B5H10	7858	23.30	14.68	−23.18
H15	5474	-	22.96	-
B5H15	7938	45.01	17.05	−25.74
H20	6016	-	22.14	-
B5H20	8409	39.78	20.16	−8.94
H25	7188	-	27.83	-
B5H25	7789	8.36	21.71	−21.99
H30	7161	-	33.90	-
B5H30	5510	−23.06	29.26	−13.69
H35	8384	-	27.00	-
B5H35	8737	4.21	34.28	26.96
H40	7055	-	28.16	-
B5H40	9065	28.49	26.77	−4.94
H45	6409	-	29.02	-
B5H45	9058	41.33	23.29	−19.75

**Table 6:** Summary of the results of the flexural test.

Specimen	Elastic Module [MPa]	Increase [%]	Flexural Strength [MPa]	Increase [%]
P	3002	-	68.05	-
H20	4047	-	46.12	-
B5H20	6066	49.89	68.92	49.44
H25	3411	-	52.93	-
B5H25	6712	96.78	52.12	−1.53
H30	4427	-	51.15	-
B5H30	7268	64.17	83.51	63.26
H35	3533	-	63.87	-
B5H35	8453	139.26	98.04	53.50
H40	4073	-	85.22	-
B5H40	8296	103.68	69.23	−18.76
H45	4897	-	76.06	-
B5H45	8255	68.57	79.55	4.59

## Data Availability

Not applicable.
